# Supplementary material for: Leather Waste Hydrolysation, Carbonization, and Microbial Treatment for Nitrogen Recovery by Ryegrass Cultivation
Source: Materials (Basel). 2024 Nov 23;17(23):5741. doi: 10.3390/ma17235741 (PMC11642390; doi:10.3390/ma17235741)
Supplement: Supplementary file 1 [file materials-17-05741-s001.zip › materials-3297868-supplementary.pdf]

Table S1. Selection of tanned leather waste-fractions used in experiments and their pre-treatments.

Hydrolysates	Symbol	Processing stage	Pretreatment procedure
1 Acids mixture used, Cu, Zn, Mn supplemented (Cr)	HC (light)	Solid waste from the leather shaving process, Cr tanned	Hydrolysis with a mixture of acids: phosphoric (V) (12%), fumaric (2%), oxalic (1.7%) and citric (1.7%) spray-method supplemented with micronutrients: Cu 0.25%, Mn 0.25% and Zn 0.25%, adjusting pH with potassium hydroxide to pH 2.5; granulation using sewage sludge
2 Acids mixture used, Cu, Zn, Mn supplemented (no Cr)	HFOC (light)	Solid waste from the leather shaving process, free of Cr	
3 Hydrochloric acid used, (Cr)	HYDC (heavy)	Residue after collagen extraction from solid waste from the leather shaving process, Cr tanned	1 M HCl extraction, L:S 3.77:1, autoclaved (121°C, 20 minutes, p = 1 bar), filtered twice, adjusted to pH 6.7 with a 25% ammonia solution
4 Hydrochloric acid used (no Cr)	HYDFOC (heavy)	Residue after collagen extraction from solid waste from the leather shaving process, free of Cr	
**Wet-green (olive extract impregnated shavings)**	**Symbol**	**Processing stage**	**Pretreatment procedure**
5 Not treated shavings (no Cr)	OLIV (wet-green)	Solid waste from the leather shaving process, where olive extract was used as impregnant free of Cr	None
6 Shavings amended with "wet white" biochar (no Cr)	OLIVb (wet-green)		Amended with a biochar obtained from the "wet white" bovine shavings (no Cr) pyrolised at 500 °C. Dose: 1 t CaO/ ha (ca. 33.7g/ pot)
7 Shavings amended with "wet white" biochar and incubated with a P-stimulating microbial consortia BactoFos (free of Cr)	OLIVb_bio (wet-green)		Amended with a biochar obtained from the "wet white" bovine shavings (no Cr) pyrolised at 500 °C incubated with a P-stimulating microbial consortia BactoFos (dilution 1:250, 2 ml/ pot used).

Table S2. Amounts of tanned leather waste fertilisers based on N content, added to the soil in the glasshouse experiment.

**Mineral Fertiliser NPK (MF)**					**"Light" Hydrolysate from bovine shavings (Cr), supplemented, granulated, (HC)**				
Dosage Nr	kg N/ ha	g N/ pot	g fertiliser/ pot	mg N/ kg soil d.m.	Dosage Nr	kg N/ ha	g N/ pot	g fertiliser/ pot	mg N/ kg soil d.m.
1 (normal)	20	0,033	0,17	0,023	1	20	0,033	3,16	0,023
2	70	0,116	0,61	0,079	2	70	0,116	11,06	0,079
3	120	0,198	1,04	0,135	3	120	0,198	18,95	0,135
4 (max in PL)	170	0,281	1,48	0,192	4	170	0,281	26,85	0,192
5	220	0,363	1,91	0,248	5	220	0,363	34,75	0,248
6	270	0,446	2,35	0,304	6	270	0,446	42,64	0,304
7	370	0,611	3,21	0,417	7	370	0,611	58,44	0,417
**"Heavy" Hydrolysate from bovine shavings (Cr), (HYDC)**					**"Heavy" Hydrolysate from bovine shavings (no Cr), (HYDFOC)**				
Dosage Nr	kg N/ ha	g N/ pot	g fertiliser/ pot	mg N/ kg soil d.m.	Dosage Nr	kg N/ ha	g N/ pot	g fertiliser/ pot	mg N/ kg soil d.m.
1 (normal)	20	0,033	14,28	0,023	1	20	0,033	12,73	0,023
2	70	0,116	49,97	0,079	2	70	0,116	44,54	0,079
3	120	0,198	85,66	0,135	3	120	0,198	76,35	0,135
4 (max in PL)	170	0,281	121,36	0,192	4	170	0,281	108,16	0,192
5	220	0,363	157,05	0,248	5	220	0,363	139,98	0,248
6	270	0,446	192,74	0,304	6	270	0,446	171,79	0,304
7	370	0,611	264,13	0,417	7	370	0,611	235,41	0,417

Table S3. Amounts of tanned leather waste fertilisers based on N content, added to the soil in the glasshouse experiment.

Wet-greens (OLIV)					Biochar (from shavings) amended Wet-greens, incubated with microbial product BactoFos (OLIV_B_bio)				
Dosage Nr	kg N/ ha	g N/ pot	g fertiliser/ pot	mg N/ kg soil d.m.	Dosage Nr	kg N/ ha	g N/ pot	g fertiliser/ pot	mg N/ kg soil d.m.
1	20	0,033	1,18	0,023	1	20	0,033	1,18	0,023
2	70	0,116	4,14	0,079	2	70	0,116	4,14	0,079
3	120	0,198	7,09	0,135	3	120	0,198	7,09	0,135
4	170	0,281	10,04	0,192	4	170	0,281	10,04	0,192
5	220	0,363	13,00	0,248	5	220	0,363	13,00	0,248
6	270	0,446	15,95	0,304	6	270	0,446	15,95	0,304
7	370	0,611	21,86	0,417	7	370	0,611	21,86	0,417

Biochar (from shavings) amended Wet-greens (OLIV_B)					"Light" Hydrolysate from bovine shavings (no Cr), supplemented, granulated, (HFOC)				
Dosage Nr	kg N/ ha	g N/ pot	g fertiliser/ pot	mg N/ kg soil d.m.	Dosage Nr	kg N/ ha	g N/ pot	g fertiliser/ pot	mg N/ kg soil d.m.
1 (normal)	20	0,033	1,181	0,023	1	20	0,033	4,633	0,023
2	70	0,116	4,135	0,079	2	70	0,116	16,215	0,079
3	120	0,198	7,089	0,135	3	120	0,198	27,797	0,135
4 (max in PL)	170	0,281	10,042	0,192	4	170	0,281	39,379	0,192
5	220	0,363	12,996	0,248	5	220	0,363	50,962	0,248
6	270	0,446	15,950	0,304	6	270	0,446	62,544	0,304
7	370	0,611	21,857	0,417	7	370	0,611	85,708	0,417

Table S4. Chemical composition of selected waste fractions and processed waste fractions for fertilisers

Element	Biochar (FOC)	HFOC	HC
**N**	10,3 ± 1,55	1,23 ± 0,18	1,97 ± 0,29
**P**	0,25 ± 0,04	8,72 ± 1,31	8,08 ± 1,21
**K**	0,04 ± 0,01	1,14 ± 0,28	1,97 ± 0,30
**S**	No Data	1,10 ± 1,17	1,05 ± 0,16
**Ca**	0,77 ± 0,12	10,0 ± 1,50	9,83 ± 1,47
**Mg**	0,04 ± 0,01	1,92 ± 0,29	1,93 ± 0,29
**Na**	3,11 ± 0,47	0,46 ± 0,07	0,39 ± 0,06
Cu	1090 ± 164	1883 ± 282	1690 ± 253
**Fe**	1470 ± 220	40510 ± 6077	39700 ± 595
**Mn**	1330 ± 200	1453 ± 217	1310 ± 196
**Mo**	<LOD (<0,055)	<LOD (<0,055)	<LOD (<0,055)
**Zn**	1205 ± 180	3858 ± 578	3610 ± 541
As	<LOD (<4,98)	<LOD (<4,98)	<LOD (<4,98)
**Ba**	<LOD (<0,02)	<LOD (<0,02)	<LOD (<0,02)
**Cd**	<LOD (<0,09)	<LOD (<0,09)	<LOD (<0,09)
**Cr (III)**	78,5 ± 11,7	77,09 ± 11,56	2680 ± 402
**Cr (VI)**	1,08 ± 0,16	No Data	0,85 ± 0,13
**Hg**	<LOD (<0,001)	<LOD (<0,001)	<LOD (<0,001)
**Ni**	<LOD (<0,56)	25,89 ± 3,88	39,8 ± 5,92
**Pb**	<LOD (<0,36)	59,92 ± 8,98	56,1 ± 8,43
Al	33840 ±5047	16520 ± 2478	14800 ± 2220
**Co**	<LOD (0,27)	<LOD (<0,27)	<LOD (0,27)
**Se**	<LOD (0,49)	<LOD (<0,49)	<LOD (0,49)

Table S5. Chemical composition of selected waste fractions and processed waste fractions for fertilisers

Material	Dry matter [%]	*Protein content [%]	*Fat content [%]	*Ash content [%]
”Heavy” hydrolyzate HYDFOC	4.42 ± 0.09	9.26 ± 0.10	0.51 ± 0.02	0.72 ± 0.05
”Heavy” hydrolyzate HYDC	5.15 ± 0.08	8.78 ± 0.12	0.36 ± 0.01	0.86 ± 0.04
